# The Heat Shock Transcription Factor HSF1 Induces Ovarian Cancer Epithelial-Mesenchymal Transition in a 3D Spheroid Growth Model

**DOI:** 10.1371/journal.pone.0168389

**Published:** 2016-12-20

**Authors:** Chase D. Powell, Trillitye R. Paullin, Candice Aoisa, Christopher J. Menzie, Ashley Ubaldini, Sandy D. Westerheide

**Affiliations:** Department of Cell Biology, Microbiology, and Molecular Biology, College of Arts and Sciences, University of South Florida, Tampa, Florida, United States of America; Boston University Medical School, UNITED STATES

## Abstract

Ovarian cancer is the most lethal gynecological cancer, with over 200,000 women diagnosed each year and over half of those cases leading to death. The proteotoxic stress-responsive transcription factor HSF1 is frequently overexpressed in a variety of cancers and is vital to cellular proliferation and invasion in some cancers. Upon analysis of various patient data sets, we find that HSF1 is frequently overexpressed in ovarian tumor samples. In order to determine the role of HSF1 in ovarian cancer, inducible HSF1 knockdown cell lines were created. Knockdown of HSF1 in SKOV3 and HEY ovarian cancer cell lines attenuates the epithelial-to-mesenchymal transition (EMT) in cells treated with TGFβ, as determined by western blot and quantitative RT-PCR analysis of multiple EMT markers. To further explore the role of HSF1 in ovarian cancer EMT, we cultured multicellular spheroids in a non-adherent environment to simulate early avascular tumors. In the spheroid model, cells more readily undergo EMT; however, EMT inhibition by HSF1 becomes more pronounced in the spheroid model. These findings suggest that HSF1 is important in the ovarian cancer TGFβ response and in EMT.

## Introduction

Ovarian cancer is the number one cause of death related to gynecological malignancies [1]. This is partially due to a lack of physical symptoms during early cancer stages as well as shortcomings in screening techniques. In fact, a majority of newly diagnosed ovarian cancer cases present with stage III and IV disease [2]. Recent advances in surgery and chemotherapy treatment have led to improvement in short-term survival of ovarian cancer patients, however long-term survival remains bleak [3]. Conventional chemotherapy agents used to treat ovarian cancer include platinum and taxol-based drugs. While these agents are largely effective upon initial treatment, the patient commonly develops resistance to the drugs, yielding them inefficient should the patient relapse [4]. In addition, agents such as cisplatin can be toxic to the patient’s organs, such as the kidneys and gastrointestinal tract, indicating a need for more efficient, as well as safer, treatment options [5].

The heat shock response (HSR), driven by the heat shock transcription factor HSF1, is a cytoprotective response to proteotoxic stressors, including heat shock, that results in the induction of various genes including molecular chaperones essential for recovery from cellular damage [6]. Chaperones function to guide protein folding and protect cells against proteotoxic stress [7]. The HSR is regulated at the transcriptional level by the heat shock transcription factor 1 (HSF1) [6].

Multiple lines of evidence suggest that HSF1 is important in promoting tumorigenesis. For instance, studies in HSF1 null mice show they are refractory to chemically-induced tumors, and HSF1 -/- mouse embryonic fibroblasts resist oncogene-induced transformation [8]. In cancer, HSF1 controls many genes that may support the transformed phenotype, including genes involved in cell-cycle regulation, signaling, metabolism, adhesion and translation [9]. HSF1 is elevated in breast, colon, lung and hepatocellular cancers, and activated or elevated HSF1 often couples with poor cancer prognosis [9, 10].

The dissemination of primary tumors occurs through a multi-step process called the epithelial-to-mesenchymal transition (EMT). EMT consists of detachment of primary tumor cells, infiltration of local stroma, spread through cavities or vascular and lymphatic vessels, and adhesion followed by colonization at distant sites [11]. Sweeping changes are made in the cytoskeleton and extracellular matrix during EMT, and cells develop a spindle-like morphology. TGFβ inhibits proliferation in normal tissues, but this effect is lost in advanced cancer where it strongly promotes EMT [12]. The expression of a number of transcription factors are induced by TGFβ and support the EMT process, including SNAI2/SLUG, SNAI1/SNAIL, TWIST1 and ZEB1 [11]. Once the mesenchymal-like cell has migrated into a new organ, it can then undergo the reverse mesenchymal-to-epithelial transition (MET) and begin to form a secondary tumor [13].

Here, we have established two ovarian cancer inducible HSF1 knockdown cell lines to study the effect of HSF1 on ovarian cancer. We show that HSF1 knockdown inhibits colony formation, wound healing, migration and the induction of FN1/fibronectin, a protein important in the EMT process. We also show that the induction of EMT markers by TGFβ is enhanced when cells are grown as 3D spheroid cultures vs. 2D monolayer cultures. Upon 3D culturing, there is a marked effect of HSF1 on the induction of transcription factors known to promote EMT. HSF1 knockdown also alters spheroid morphology. Thus, we conclude that HSF1 plays a striking role in regulating the EMT process under 3D growth conditions.

## Materials and Methods

### HSF1 copy number, expression determination and survival analysis

Data comparing HSF1 copy number across multiple cancers with GISTIC analysis was obtained from The Cancer Genome Atlas (TCGA) via the cBio portal [14, 15]. HSF1 expression levels across multiple cancers were assessed from TCGA RNA seq V2 data via the cBio portal. Data for the comparison of ovarian cancer and normal ovarian tissue were obtained from GEO and the TCGA. The datasets analyzed were: GSE18520, consisting of 10 normal ovary and 53 ovarian cancer samples assayed on Affymetrix HG-U133 Plus 2.0 GeneChips, and TCGA data, consisting of 8 normal ovary and 568 ovarian cancer samples assayed on Affymetrix HG-U133A GeneChips. Gene intensity was compared by one-sided unpaired T-test.

### Cell culture and treatments

HEY, SKOV3 and T80 cells were authenticated using short tandem repeat (STR) DNA profiling (Genetica, Inc.) and comparing profiles to ATCC profiles and other previously published profiles [16]. Cells were cultured in RPMI 1640 medium supplemented with 10% fetal bovine serum (GIBCO) and 1% Pen-Strep-Glutamine (CellGro) in a humidified incubator at 37°C with 5% CO_2_. Heat shock treatment was performed by wrapping plates in parafilm and submerging them in a 42°C circulating water bath for designated times. Cells were treated as indicated with 1 μg/ml doxycycline (Sigma-Aldrich) and 5 ng/mL TGFβ1 (Thermo Fisher).

### Lentiviral creation and infection for stable, inducible shRNA-mediated HSF1 knockdown

To allow for inducible knockdown of HSF1, we utilized the doxycycline-inducible TRIPZ shRNAmir system (Thermo Scientific). Two shRNA sequences targeting HSF1 were obtained from the RNAi codex database [17]: CGCAGCTCCTTGAGAACATCAA (shHSF1A) and CCCACAGAGATACACAGATATA (shHSF1B). These two sequences were cloned into the pTRIPZ vector. For lentiviral packaging, a 2^nd^ generation lentiviral system was used with the pCGP packaging and pVSVG envelope plasmids (Addgene). HEK293T cells, cultured in RPMI medium, were used as the packaging cell line. Transfection was achieved using Polyfect Transfection Reagent (Qiagen) according to the manufacturer’s protocol using a 1:1:1 ratio of lentiviral vectors. 24 hours post-transfection, medium with transfection reagent was removed and replaced with fresh RPMI. Medium containing viral stock from the HEK293T cells was harvested 48 hours post-transfection. A 0.45 micron PVDF filter was used to filter viral stock and infection of the HEY and SKOV3 cell lines was performed in a single round with the addition of 8 μg/mL of hexadimethrinebromide (Sigma-Aldrich). Selection of stable HEY and SKOV3 cells was achieved with 1 μg/ml and 0.5 μg/ml of puromycin (Thermo Fisher) for the HEY and SKOV3 cell lines, respectively. Infection was verified via immunoblotting analysis for knockdown of HSF1 after doxycycline-induced expression of the shRNAs.

### Protein isolation, SDS-PAGE, and western analysis

Cells were washed once and scraped in chilled PBS. After pelleting the cells, protein was extracted using the M-PER lysis buffer (Thermo Scientific) with a protease inhibitor cocktail (Halt^™^ Protease Inhibitors, Thermo Scientific). A Bio-Rad Protein Assay was then utilized to quantify protein concentrations. 20 μg of lysate was resolved on 8% to 12% sodium dodecyl sulphate polyacrylamide gel electrophoresis (SDS-PAGE) gels and transferred to Immun-Blot^®^ 0.2μm PVDF Membrane with a Trans-Blot semi-dry transfer cell (Bio-Rad). Membranes were blocked in 2% w/v non-fat milk in TBS with 0.1% Tween (TBST milk). Blots were probed with primary and secondary antibodies before incubation in ECL Prime Western Blotting Detection System (Amersham^™^) and film exposure. Primary antibodies used were: HSF1 (Assay Designs), HSF1 P-S326 (Abcam) fibronectin (BD Biosciences), HSP90 (Cell Signaling), HSP70 (Cell Signaling) and Actin (Santa Cruz). HRP-conjugated secondary antibodies were from Millipore and Jackson ImmunoResearch.

### Cell viability assay

Cells at a concentration of 2 x 10^5^ cells/ml were seeded in a 96-well plate at 100 μl per well with eight replicates for each test condition. The cells were then incubated either with or without doxycycline treatment for 72 hours. After incubation, 10 μl of PrestoBlue Cell Viability Reagent (Invitrogen) was added to each well and incubated for 1 hour at 37°C. The reduction of the reagent was measured by fluorescence (excitation 570 nm, emission 600 nm) using a microplate reader (BioTek). Mean percent viability and standard error were then plotted.

### Clonogenic assay

Cells were seeded at 500 cells per well in 6-well plates and were treated with or without 1 μg/ml doxycycline to induce HSF1 knockdown. Treated wells were given an additional treatment with 1 μg/ml doxycycline on day 4 to maintain doxycycline levels. After 8 days, colonies were stained with 1% crystal violet (w/v) in methanol and rinsed 3X in deionized water. Stained colonies were subsequently photographed and counted.

### Wound healing assay

Cells were plated at 3 x 10^5^ cells per well in a 6-well plate, and then either treated with 1 μg/ml doxycycline 48 hours prior to the assay or left untreated. Once the cells reached confluency, a 2 μl pipet tip was used to scrape the cells in 2 vertical and 2 horizontal lines yielding 4 intersections per well. Cells were washed twice with PBS to remove debris and serum-free medium was added. Pictures were taken immediately and again 12 hours after the creation of the wound, using an EVOS inverted microscope (Advanced Microscopy Group). The experiment was performed in triplicate and wound closure was determined using TScratch software [18]. Significant differences were calculated by ANOVA and Bonferroni post-hoc tests.

### Cell migration

For the transwell migration assay, cells were treated with or without 1 μg/ml doxycycline 48 hours prior to the assay to induce HSF1 knockdown, and cells were then serum-starved 24 hours before performing the assay. Cells were then resuspended in serum-free medium, and seeded at 2.5 x 10^4^ cells per upper chamber. 400 μL of complete medium containing FBS was added as a chemoattractant to the lower chamber. After a 16-hour incubation, non-migrating cells on the upper surface of the filter were removed by scrubbing with a cotton swab. The remaining cells on the lower surface were fixed and stained with 1% (w/v) crystal violet in methanol. Migrated cells were counted from 10 random fields of view from each well and each condition was performed with triplicate samples. Statistical analysis done by paired t-test.

### Spheroid formation

The hanging drop method was utilized to form spheroids [19]. Briefly, cells released with trypsin were resuspended at 1 X 10^6^ cells/mL in RPMI medium, supplemented as described above. Cell suspension droplets of 25 μl were placed on the plate lids, which were then inverted and put back on plates containing phosphate buffered saline (PBS) and incubated for 48 hours. Upon incubation, cells aggregated into spheroids. Prior to plating the cells, TGFβ1 was added to the suspension as indicated at a final concentration of 5 ng/mL. Following aggregation for 48 hours, spheroids were collected in 1X PBS. Pictures were obtained using an EVOS (Advanced Microscopy Group) inverted microscope.

### Quantitative RT-PCR

Cells were harvested in cold 1X PBS and RNA extraction was completed utilizing the TRIzol reagent (Thermo Fisher) according to standard protocol. Reverse transcription reactions of the RNA were performed with the High Capacity cDNA Reverse Transcription Kit (Applied Biosystem), as per the manufacturer’s protocol. The cDNA samples were then used as a template for qRT-PCR. Applied Biosystem’s Step One Plus Real-time PCR machine was used with BioRad’s iTaq^™^ Fast SYBR^®^ Green Supermix with ROX according to the manufacturer’s protocol. The primer sets used for each gene can be found in S1 Table. GAPDH was used as the endogenous reference control. Statistical significance was measured by Student’s t test.

## Results

### HSF1 is overexpressed in ovarian cancer

**Fig 1:**
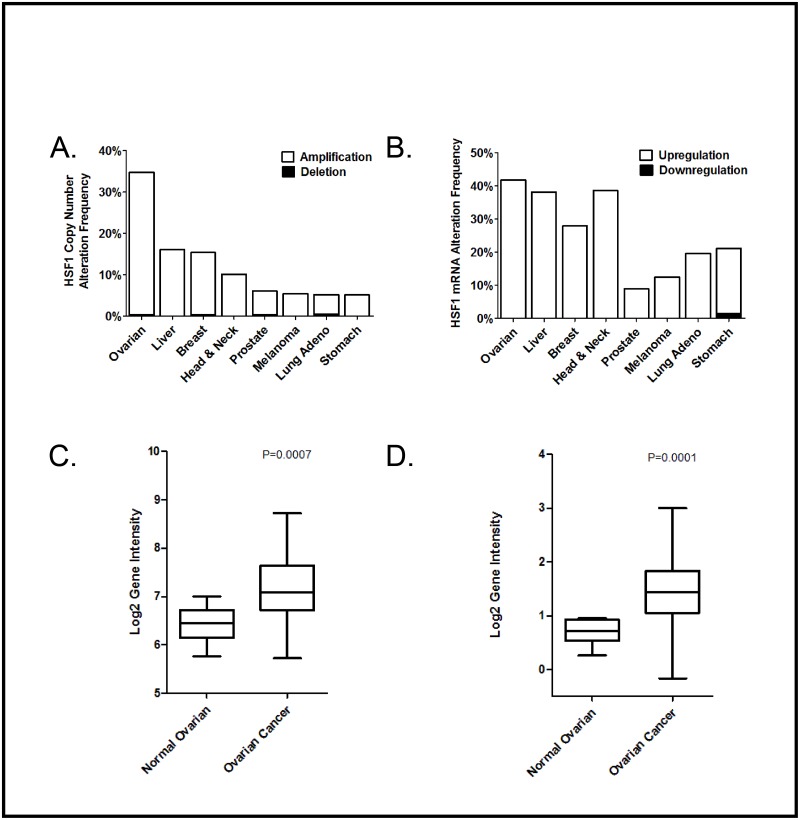
HSF1 levels are elevated in ovarian cancer patient samples. A, HSF1 copy number is increased most frequently in ovarian cancer. HSF1 copy number was analyzed in a variety of cancers using TCGA data and GISTIC analysis with a threshold CNA change of +/-2. B, HSF1 transcripts are elevated in a variety of cancers. Samples from tumor tissue and matched normal tissue were compared in the TCGA database using RNA Seq V2 RSEM data with a z-score threshold of +/-2. C, HSF1 is increased at the mRNA level in an ovarian cancer data set GSE18520 consisting of 10 normal ovarian samples and 53 late stage, primary site, high grade ovarian cancer samples. D, HSF1 is increased at the mRNA level in a TCGA ovarian cancer data set consisting of 8 normal ovarian samples and 568 ovarian cancer samples.

We analyzed data from The Cancer Genome Atlas (TCGA) database to compare HSF1 levels across multiple cancer types. Interestingly, we find that HSF1 gene duplication is more common in ovarian cancer than in any other cancer type in this database by a large margin (Fig 1A). Additionally, we find that HSF1 mRNA transcripts are elevated in ovarian cancer tumor tissue vs. normal epithelial tissue from matched patient samples (Fig 1B). Other cancers with high HSF1 mRNA levels include liver cancer, head and neck cancer, and breast cancer (Fig 1B). Two distinct data sets of matched ovarian tumor tissue vs. normal tissue show that HSF1 mRNA expression is significantly higher in tumor tissue (Fig 1C and 1D). Given this data, we postulate that HSF1 may drive ovarian cancer progression. We thus sought to study the effect of HSF1 knockdown in ovarian cancer cell lines.

### Establishment of SKOV3 and HEY inducible HSF1 knockdown ovarian cancer cell lines

**Fig 2:**
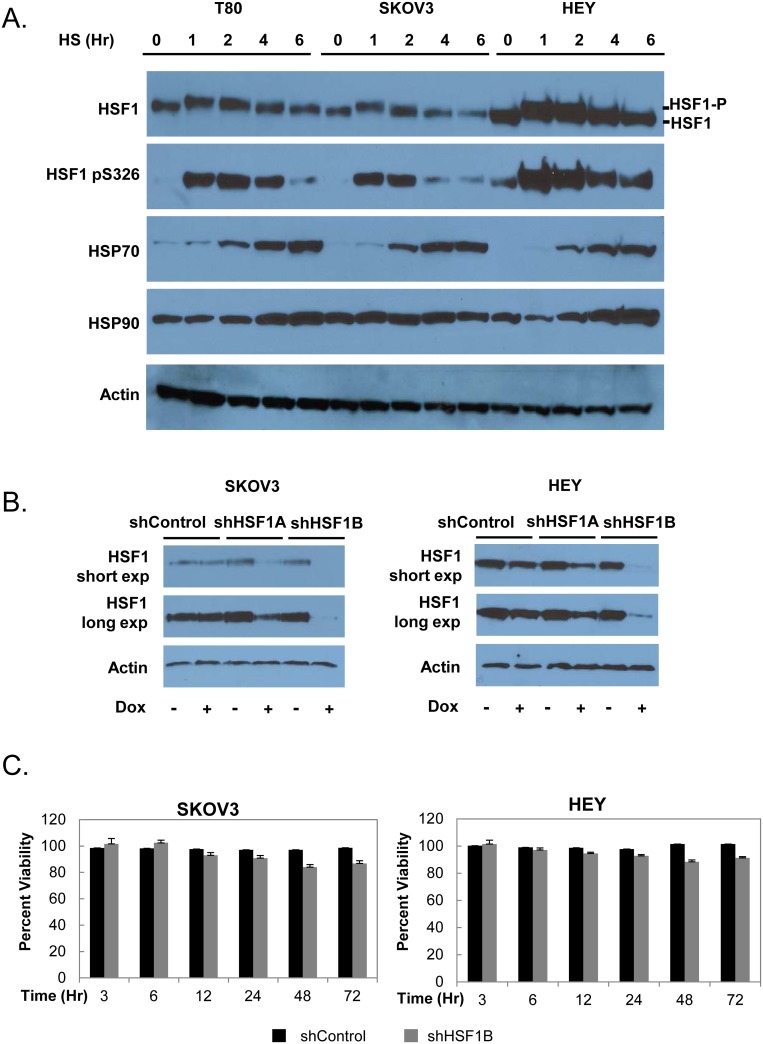
Validation of inducible HSF1 knockdown ovarian cancer cell lines. A, The heat shock response in the epithelial ovarian carcinoma cell lines SKOV3 and HEY as compared to normal ovarian epithelial T80 cells. T80, SKOV3, and HEY cells were treated with a 42°C heat shock for the indicated times and harvested immediately after. Cell lysates were subjected to Western blot analysis using antibodies recognizing HSF1, HSF1 phosphorylated at S326, HSP90, HSP70, and actin as a loading control. B, The pTRIPZ system was used to create the doxycycline-inducible HSF1 knockdown cell lines SKOV3.shHSF1A, SKOV3.shHSF1B, HEY.shHSF1A and HEY.shHSF1B. After treatment with 1 μg/ml doxycycline for 48 hours, cell lysates were subjected to Western blot analysis using antibodies recognizing HSF1 and actin as a loading control. Both short and long exposures are shown for the HSF1 blot. C, HSF1 knockdown does not cause a large decrease in cell viability. The viability of the SKOV3.shHSF1B and HEY.shHSF1B cell lines as compared to shControl cells was assessed after treatment with 1 μg/ml doxycycline for the indicated times using the PrestoBlue cell viability assay. Mean percent viability (n = 8) and standard error is shown.

We chose two epithelial ovarian cancer cell lines for our studies, SKOV3 and HEY. These cell lines were authenticated by using short tandem repeat (STR) DNA profiling (Genetica, Inc.) and comparing the profiles to ATCC profiles and other previously published profiles [16]. We first wanted to test whether the cell lines we selected exhibited a normal response to heat, including the characteristic activation of HSF1 and induction of chaperones. We find that both SKOV3 and HEY cells exhibit multiple hallmarks of activation of the heat shock response (Fig 2A). Upon treatment with a 42°C heat shock over a 6 hour timecourse, we observe stress-induced hyperphosphorylation of HSF1 followed by a return to the hypophosphorylated state. This result is characteristic of HSF1 activation by heat shock and can be readily detected by electrophoretic retardation on SDS-PAGE and Western blot analysis [20]. Interestingly, while SKOV3 cells contain a similar level of basal and activated HSF1 as compared to normal ovarian epithelial T80 cells, HEY cells express higher levels of HSF1, corresponding to the higher levels of HSF1 expression we identified in ovarian cancer patient databases. Upon heat shock, we also observed that both SKOV3 and HEY cells show HSF1 phosphorylation at serine 326, a marker of activated HSF1 [21]. Additionally, the chaperone HSP70 was induced by heat shock in both SKOV3 and HEY cells, and HSP90 was induced in HEY cells. Overall, we conclude that both SKOV3 and HEY cells express HSF1 and respond to heat shock, validating the choice of these two ovarian cancer cell lines for our studies.

We next wanted to generate HSF1 knockdown SKOV3 and HEY cell lines. Our initial attempts to create stable HSF1 knockdown in these cell lines were not successful, perhaps due to selective pressure for the cancer cells to re-express HSF1. We therefore employed a doxycycline-inducible shHSF1 system (pTRIPZ vector, Open Biosystems). To ensure that doxycycline treatment alone would not alter HSF1 levels or activity, we treated SKOV3 and HEY cells with both 0.5 and 2.0 μg/ml of doxycycline for 48 hours and found no changes in HSF1 levels or hyperphosphorylation status (S1 Fig). We also found no change in HSP90 levels (S1 Fig). We therefore concluded that a doxycycline-inducible system would be a viable option for HSF1 knockdown in our studies.

We used two shHSF1 sequences obtained from the RNAi codex database [17], shHSF1A (CGCAGCTCCTTGAGAACATCAA) and shHSF1B (CCCACAGAGATACACAGATATA), as well as a control sequence that is non-targeting, to create SKOV3.shControl, SKOV3.shHSF1A, SKOV3.shHSF1B, HEY.shControl, HEYshHSF1A and HEY.shHSF1B stable cell lines (Fig 2B). The shHSF1A sequence knocks down HSF1 expression by about 75%, while shHSF1B knocks down HSF1 expression more completely. Knockdown of HSF1 resulted in only a marginal reduction of cell viability in the SKOV3 or HEY cell lines over a 72 hour doxycycline treatment timecourse (Fig 2C). We thus have established an effective means of knocking down HSF1 to varying degrees in two different ovarian cancer cell lines.

### HSF1 knockdown inhibits colony formation, wound healing, cell migration and fibronectin expression

**Fig 3:**
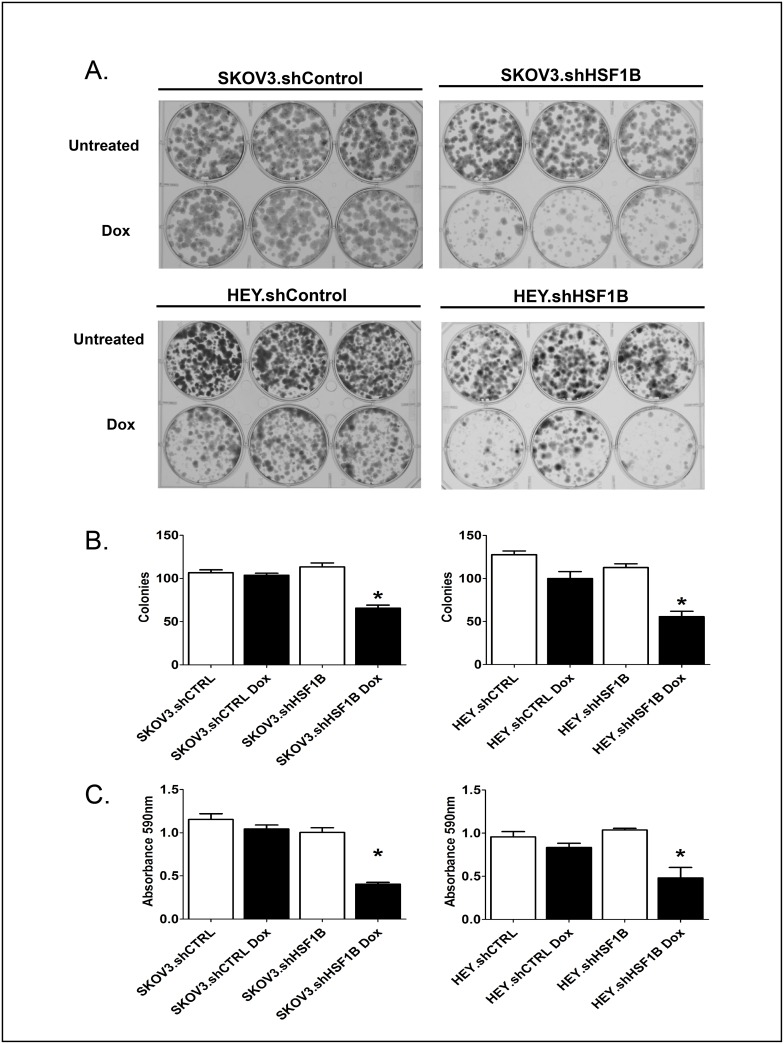
HSF1 knockdown reduces colony formation. SKOV3.shHSF1B, HEY.shHSF1B and control cell lines were plated 250 cells per well in 6-well plates in triplicate. Cell were treated with or without 1 μg/ml doxycycline (Dox) to induce HSF1 knockdown and were given an additional dose after 4 days. Cells were stained with crystal violet after 8 days to visualize colonies.

We then assayed our HSF1 knockdown cell lines to determine whether HSF1 is important for ovarian cancer tumorigenicity. As a measure of the ability of HSF1 to allow cell survival and growth upon plating at a low cell density, clonogenic assays were performed (Fig 3). SKOV3.shControl, SKOV3.shHSF1B, HEY.shControl and HEY.shHSF1B stable cells were treated with or without doxycycline to induce HSF1 knockdown and then plated at 250 cells per well in 6-well plates in triplicate. Colonies, stained after 8 days, show that HSF1 knockdown strongly inhibits colony formation in both HEY and SKOV3 cells.

**Fig 4:**
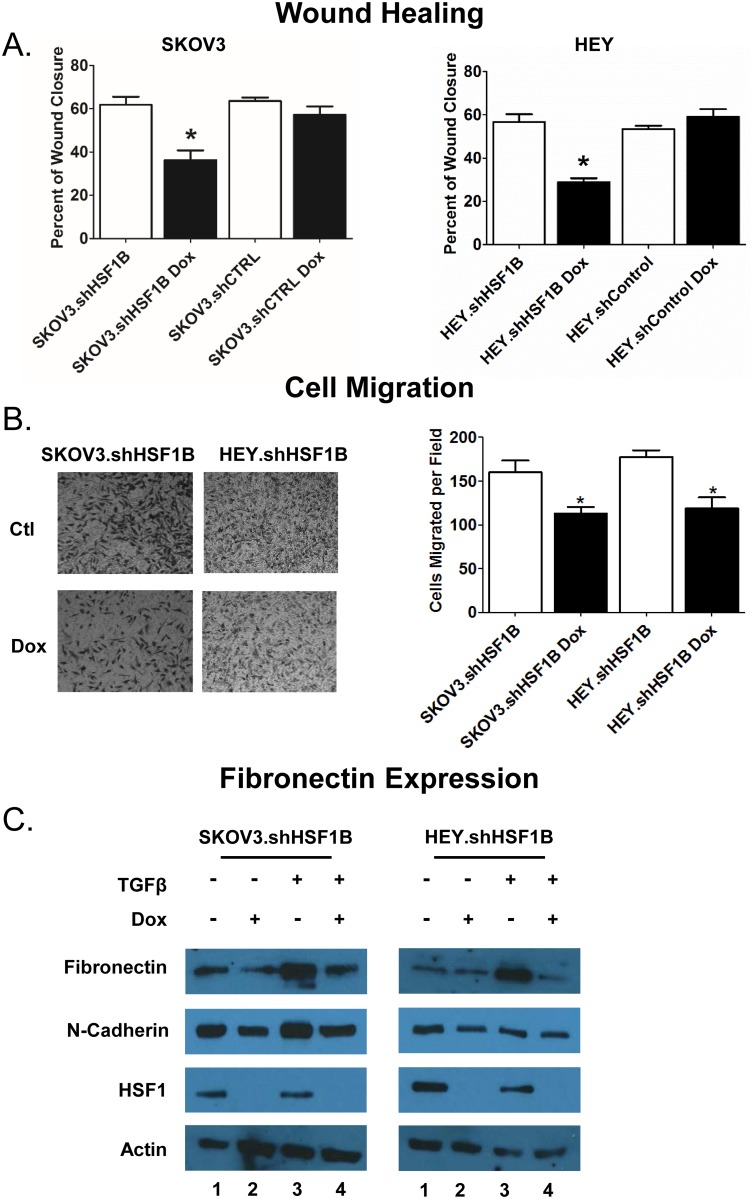
HSF1 knockdown inhibits wound healing, migration and induction of fibronectin. A, HSF1 knockdown reduces wound closure. Cells treated with or without 1 μg/ml doxycycline were grown in 6-well plates to confluency. Cells were scraped to create wounds, the cells were washed and serum-free media was added. The intersections of perpendicular scratches were photographed immediately and 12 hours after and analyzed using Tscratch software. Asterisk denotes significant difference from all other samples calculated by ANOVA (P <0.05). B, HSF1 knockdown reduces migration. After treatment with or without 1 μg/ml doxycycline and 12 hours of serum starvation, cells were added to a Boyden chamber at 2.5 x 10^4^ cells per chamber. Serum was used as the chemoattractant in the lower chamber. After 16 hours, nonmigrating cells were scrubbed and cells which had migrated stained. The experiment was done in triplicate and analysis done by paired t-test. Asterisk marks significant difference (P < 0.05). C, HSF1 KD reduces TGFβ-induced expression of fibronectin. SKOV3.shHSF1B and HEY.shHSF1B were treated with 1ug/ml doxycycline, 10 ng/μl TGFβ, or both, and cell lysates were harvested for immunoblotting. Cell lysates were subjected to Western blot analysis using antibodies recognizing fibronectin, HSF1, and actin as a loading control.

To assess the ability of HSF1 to affect cellular motility, we used a wound healing assay as well as a cell migration assay. For the wound healing assay, cells were seeded in equal numbers into 6-well plates and grown to ~80% confluence prior to introducing scratches in straight lines through the monolayers. TScratch software was then used to automatically analyze wound healing rates (Fig 4A). HSF1 knockdown in SKOV3 and HEY cells inhibits wound-healing ability by 25% and 28%, respectively. Next, cell migration assays were employed to assess the ability of cells to pass through a matrigel-coated transwell membrane (Fig 4B). Cells were seeded in equal numbers into the insert of a transwell plate, with no cells in the lower chamber. The number of cells that passed through the membrane were then calculated and plotted after 48 hrs. HSF1 knockdown was found to inhibit cell migration by 29% in SKOV3 cells and 33% in HEY cells. These experiments in sum support a role for HSF1 in promoting cell motility in ovarian cancer.

We next wanted to test whether HSF1 knockdown can suppress the EMT process. Fibronectin, a mesenchymal marker, is upregulated during EMT and plays a crucial role in altering cell adhesion and migration processes, allowing for transition to the mesenchymal state [22]. We tested protein expression levels of fibronectin using Western blot analysis of SKOV3.shHSF1B and HEY.shHSF1B cells treated with and without doxycycline and with and without the EMT inducer TGFβ (Fig 4C). As expected, TGFβ treatment induces fibronectin expression (Fig 4C, compare lanes 1 with lanes 3). Interestingly, HSF1 knockdown in both SKOV3 and HEY cells reduces both the basal expression levels of fibronectin (Fig 4C, compare lanes 1 and 2) as well as the TGFβ-induced levels of fibronectin (Fig 4C, compare lanes 3 and 4). Therefore, HSF1 may promote the EMT process by enhancing TGFβ-induced fibronectin expression.

### The induction of fibronectin by TGFβ is enhanced in 3D cultures as compared to 2D cultures

**Fig 5:**
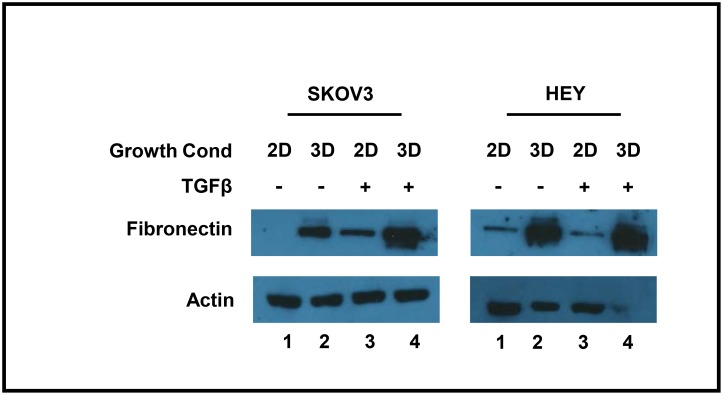
Fibronectin expression is induced by 3D growth. SKOV3 and HEY cells were cultured under 2D or 3D conditions, with or without TGFβ, as indicated. Cell lysates were subjected to Western blot analysis using antibodies recognizing fibronectin, and actin as a loading control.

As ovarian cancer cells typically spread throughout the peritoneal cavity in the form of 3D spheroids, we cultured cells in 3D culture using the hanging drop method [23] in order to create a more biologically-relevant *in vitro* system for our studies. We first tested whether the induction of fibronectin by TGFβ is altered in 3D cultures as compared to 2D cultures. In both monolayer and spheroid SKOV3 cells, TGFβ increased fibronectin expression (Fig 5). Surprisingly, this effect was enhanced in the SKOV3 spheroid model as compared to monolayer cells (Fig 5, compare lanes 2 and 4). The HEY cells also showed enhanced fibronectin expression upon 3D growth, although this effect was not enhanced by TGFβ. Therefore, we conclude that 3D culturing enhances fibronectin expression.

### 3D culturing reveals a marked effect of HSF1 on the induction of EMT transcription factors

**Fig 6:**
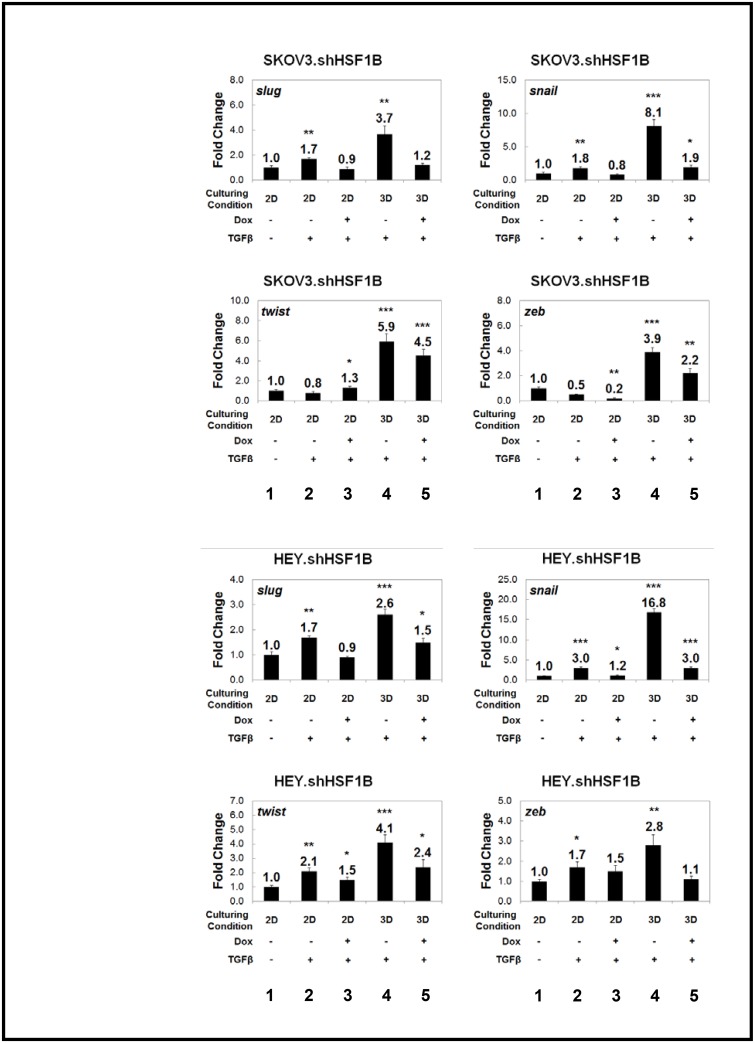
TGFβ induction of EMT master-switch transcription factors are reduced upon HSF1 knockdown, and the effect is enhanced upon 3D culturing. Quantitative real-time polymerase chain reaction (qRT-PCR) of selected genes shows that the EMT master-switch transcription factors *SNAI1/SNAIL*, *TWIST1*, *ZEB1*, *and SNAI2/SLUG* are upregulated when HSF1 inducible knockdown SKOV3.shHSF1B and HEY.shHSF1B cells are cultured as 3D spheroids. This effect is significantly reduced upon knockdown of HSF1 via doxycycline treatment. Gene expression was normalized to the housekeeping gene *GAPDH*, and fold change was calculated relative to monolayer non-treated conditions. Statistical significance was measured by Student’s t test as compared to untreated monolayer cell culture (*p<0.05; **p<0.01; ***p<0.001).

Various transcription factors, including snail, slug, twist, and zeb, help to coordinate the EMT process [11]. We tested whether 3D growth affected the expression of these genes (Fig 6). We find that 3D growth enhances TGFβ induction of these transcription factors as shown by qRT-PCR (Fig 6, compare lanes 2 and 4). We wondered whether HSF1 may regulate the expression of these EMT transcription factors. We thus tested our HSF1 knockdown cell lines, grown under both 2D and 3D conditions, to test for effects on the expression of *SNAIL*, *TWIST1*, *SLUG* and *ZEB1* mRNAs (Fig 6). SKOV3.shControl, SKOV3.shHSF1B, HEY.shControl, and HEY.shHSF1B stable cell lines, grown both as 2D and 3D cultures, were treated with and without doxycycline treatment to induce HSF1 knockdown. We find that HSF1 knockdown in most cases slightly inhibits the expression of EMT transcription factors in SKOV3 and HEY cells grown in 2D (Fig 6, compare lanes 2 with lanes 3). Interestingly, the effect of HSF1 knockdown on the expression of these genes is magnified for most of the genes upon growth in 3D conditions (Fig 6, compare lanes 4 with lanes 5). Therefore, using a 3D ovarian cancer culturing system, we have uncovered a positive effect of HSF1 on the ability of TGFβ to induce EMT genes. We thus conclude that HSF1 promotes EMT in ovarian cancer 3D spheroids at least in part through regulating the levels of EMT-inducing transcription factors.

## Discussion

As ovarian cancer is highly lethal and has few treatment options, identifying new therapeutic targets for this disease is highly important. Through mining patient data, we find that HSF1 DNA levels are most highly amplified in ovarian cancer as compared to other cancers, and also that ovarian cancer is one of the top cancer types with amplified HSF1 mRNA levels. A previous study of 37 malignant vs. benign ovarian tumors has shown that HSF1 expression is higher in the malignant tumors [24]. Our findings thus add to this data and suggest that HSF1 may be an important therapeutic target for ovarian cancer.

We have identified HSF1 as a critical player in promoting ovarian cancer tumorigenicity by multiple measures in both SKOV3 and HEY ovarian cancer cells. Via HSF1 knockdown and colony formation assays, we show that HSF1 promotes the ability of cells to grow under conditions of low cell density, a hallmark of cancer cells. Cell motility is another characteristic of cancer cells. Previous work has shown that cell motility is inhibited in immortalized mouse embryonic fibroblast cells derived from *hsf1* -/- mice [25]. In addition, HSF1 knockdown reduces the invasiveness of multiple types of tumor cells [10, 26–29]. Consistent with these findings, we show that HSF1 knockdown inhibits wound healing and cell migration in SKOV3 and HEY ovarian cancer cell lines. Our results thus add further evidence that HSF1 enhances tumorigenicity in multiple types of cancer.

EMT is essential for cell migration and is a key rate-limiting step in metastasis. Previous studies have shown that HSF1 promotes EMT in breast cancer cells through a mechanism that requires HER2 [30, 31]. As ovarian cancer cells typically spread throughout the peritoneal cavity in the form of 3D spheroids [32], culturing ovarian cancer cells as spheroids is likely to better mimic the *in vivo* growth conditions as compared to conventional 2D culturing conditions. Here, we show that HSF1 knockdown reduces the ability of TGFβ to induce EMT. Interestingly, we find that this effect is stronger upon growth in 3D spheroids. We also show that HSF1 is required for the compact morphological structure of spheroid growth.

Our data suggests that HSF1, either directly or indirectly, controls the expression of transcription factors that are important for the EMT process. Interestingly, upon promoter analysis, we find consensus heat shock element (HSE) sequences containing three inverted arrays of the sequence nGAAn [33] in the promoters of the EMT transcription factor genes *SNAIL*, *ZEB* and *TWIST1* (S2 Table). Putative HSEs are also present in the *FN1* (*fibronectin*), *VIM* (*vimentin*), *and CDH2* (*N-cadherin*) promoters, additional genes that are associated with EMT (S2 Table). Future experiments will be required to determine whether any of these genes are direct HSF1 targets. This is plausible given that HSF1 was recently found to bind to the *SLUG* promoter through an imperfect HSE motif [30].

In summary, we have identified HSF1 as a critical player in ovarian cancer progression, and have identified EMT as a process that is promoted by HSF1. The effects for HSF1 are more striking when cells are grown as 3D spheroids, which more closely mimic the *in vivo* growth conditions of ovarian cancer. Therefore, HSF1 deserves further research and development as a promising anticancer strategy for ovarian cancer.

## Supporting Information

S1 Fig Doxycycline treatment alone does not alter HSF1 levels or induce HSP90 expression in ovarian cancer cell lines. SKOV3 and HEY cells were treated with 0–2 μg/ml doxycycline, as indicated, for 48 hours. Cell lysates were subjected to Western blot analysis using antibodies recognizing HSF1, HSP90, and actin as a loading control.
(TIF)

S1 Table List of primers used in quantitative RT-PCR. (DOCX)

S2 Table Locations of HSEs in EMT genes. (DOCX)
