# Supplementary figures and images for: The Heat Shock Transcription Factor HSF1 Induces Ovarian Cancer Epithelial-Mesenchymal Transition in a 3D Spheroid Growth Model
Source: PLoS One. 2016 Dec 20;11(12):e0168389. doi: 10.1371/journal.pone.0168389 (PMC5172610; doi:10.1371/journal.pone.0168389)

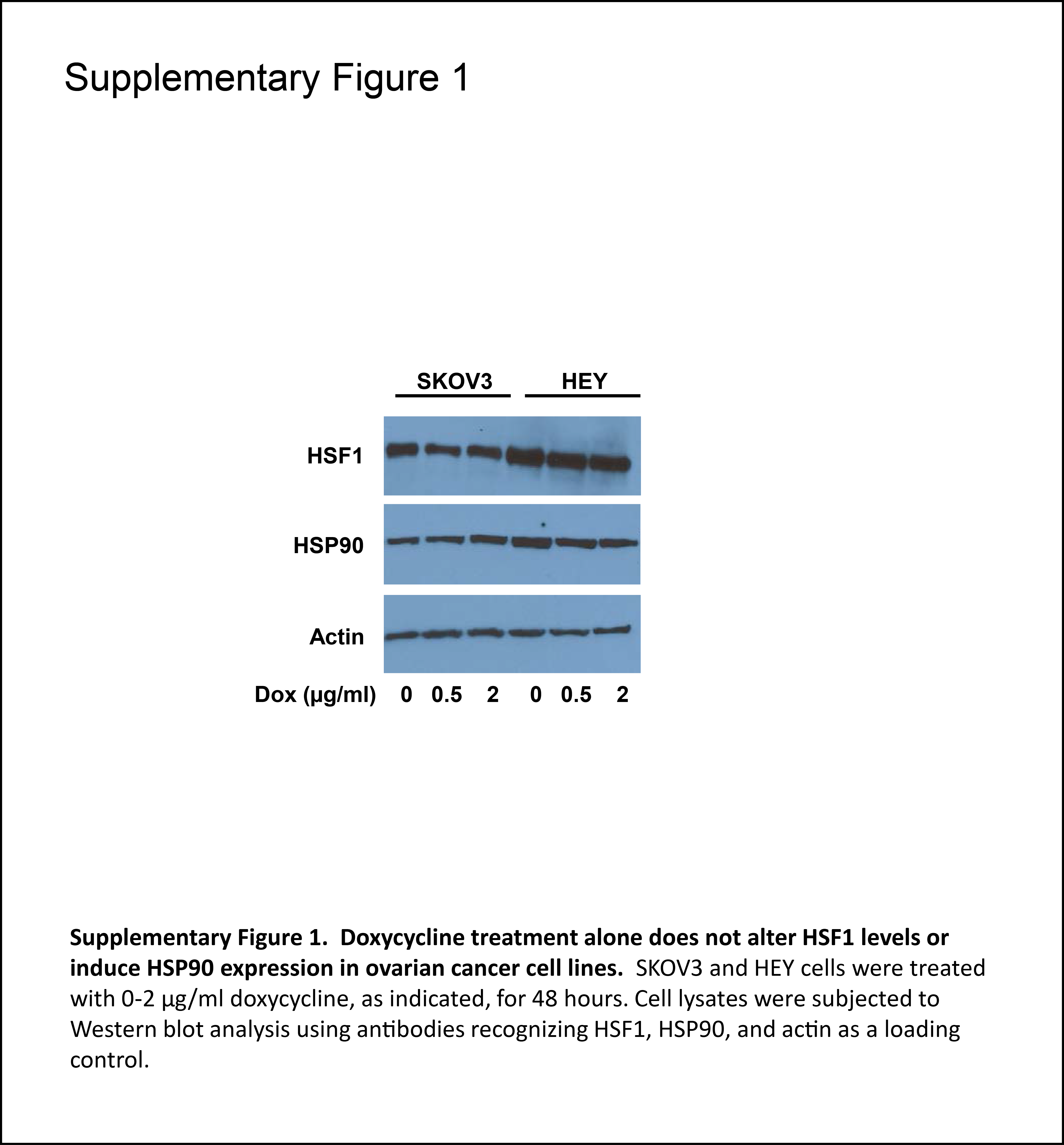

Supplement: S1 Fig — SKOV3 and HEY cells were treated with 0–2 μg/ml doxycycline, as indicated, for 48 hours. Cell lysates were subjected to Western blot analysis using antibodies recognizing HSF1, HSP90, and actin as a loading control. (TIF) [file pone.0168389.s001.tif]
