# Supplementary material for: The Heat Shock Transcription Factor HSF1 Induces Ovarian Cancer Epithelial-Mesenchymal Transition in a 3D Spheroid Growth Model
Source: PLoS One. 2016 Dec 20;11(12):e0168389. doi: 10.1371/journal.pone.0168389 (PMC5172610; doi:10.1371/journal.pone.0168389)
Supplement: S1 Table — (DOCX) [file pone.0168389.s002.docx]

**S1 Table. List of primers used in Quantitative RT-PCR.**

| **Gene Name** | **Common Name** | **Sequence** |
| --- | --- | --- |
| *GAPDH* | *GAPDH* | F: 5’ - CCACTCCTCCACTTTGAC - 3’  R: 5’ - ACCCTGTTGCTGTAGCCA - 3’ |
| *SNAI1* | *SNAIL* | F: 5’ - TCCCAGATGAGCATTGGCAG - 3’  R: 5’ - CGCGCTCTTTCCTCGTCAG - 3’ |
| *SNAI2* | *SLUG* | F: 5’ - TTCGGACCCACACATTACCT - 3’  R: 5’ - GCAGTGAGGGCAAGAAAAAG - 3’ |
| *TWIST1* | *TWIST* | F: 5’ - GGAGTCCGCAGTCTTACGAG - 3’  R: 5’ - TCTGGAGGACCTGGTAGAGG - 3’ |
| *ZEB1* | *ZEB* | F: 5’ - CAATACCGTCATCCTCAGCA - 3’  R: 5’ - CCAATCCCAGGAGGAAAAAC - 3' |
