# Supplementary material for: The Heat Shock Transcription Factor HSF1 Induces Ovarian Cancer Epithelial-Mesenchymal Transition in a 3D Spheroid Growth Model
Source: PLoS One. 2016 Dec 20;11(12):e0168389. doi: 10.1371/journal.pone.0168389 (PMC5172610; doi:10.1371/journal.pone.0168389)
Supplement: S2 Table — (DOCX) [file pone.0168389.s003.docx]

**S2 Table. Location of HSEs in EMT genes.**

| **Gene Name** | **Common Name** | **Location from CDS** | **Sequence** |
| --- | --- | --- | --- |
| *FN1* | *fibronectin* | -3254 | **TTC**TGC**AA**CT**TTC**A |
| *VIM* | *vimentin* | -3754 | **TTC**CA**GAA**GG**TT**AA |
| *SNAI1* | *SNAIL* | -3201 | **TTC**TA**GAA**GC**TTC**A |
|  |  | -3207 | **TTC**TA**GAA**TT**TT**GG |
| *CDH2* | *N-cadherin* | -4429 | **TTC**TG**G**G**A**AG**TTC**C |
|  |  | -2183 | **TTC**CG**GAA**CC**TT**TT |
|  |  | -2177 | **TTC**CG**GAA**AA**TT**TA |
|  |  | -2544 | **TTC**CT**G**G**A**TT**TTC**T |
| *ZEB1* | *ZEB* | -289 | **TTC**ACT**AA**CT**TTC**C |
| *TWIST1* | *TWIST* | -1301 | **TTC**GA**G**C**A**CC**TTC**C |
| Consensus |  |  | **TTC**nn**GAA**nn**TTC**n |
